# Interatrial septum dissection and closure from transseptal puncture during mitral transcatheter edge-to-edge repair: a case report

**DOI:** 10.1093/ehjcr/ytae559

**Published:** 2024-11-02

**Authors:** Isabel Kim, Xi E Li, Pascal T Lauzier, Rahul P Sharma, Christiane Haeffele

**Affiliations:** Division of Cardiovascular Medicine, Department of Medicine, Stanford University School of Medicine, CVRC Falk, 300 Pasteur Drive, Stanford, CA 94305, USA; Department of Anesthesia, Stanford University School of Medicine, 300 Pasteur Drive, Stanford, CA 94305, USA; Division of Cardiovascular Medicine, Department of Medicine, Stanford University School of Medicine, CVRC Falk, 300 Pasteur Drive, Stanford, CA 94305, USA; Division of Cardiovascular Medicine, Department of Medicine, Stanford University School of Medicine, CVRC Falk, 300 Pasteur Drive, Stanford, CA 94305, USA; Division of Cardiovascular Medicine, Department of Medicine, Stanford University School of Medicine, CVRC Falk, 300 Pasteur Drive, Stanford, CA 94305, USA

**Keywords:** Transseptal puncture complications, Interatrial dissection, Chronic steroid use considerations for transcatheter procedures, Case report, Transcatheter edge-to-edge repair

## Abstract

**Background:**

An 85-year-old woman was referred for mitral valve transcatheter edge-to-edge repair (TEER) following multiple heart failure hospitalizations related to her severe functional mitral regurgitation. In addition to previous transcatheter aortic valve replacement for aortic stenosis, her past medical history was significant for chronic steroid use related to microscopic polyangiitis leading to renal transplantation.

**Case summary:**

Her procedure was complicated by an interatrial septum dissection with associated haemopericardium originating from the transseptal puncture site. To our knowledge, our case demonstrates the first use of an Amplatzer occluder device to manage this complication by preventing ongoing flow into the pericardial space. This complication occurred in the setting of a single, successful transseptal puncture in the typical location for a mitral valve TEER procedure, raising the consideration that chronic steroid use may be an underappreciated risk factor for iatrogenic interatrial septal damage.

**Discussion:**

Transoesophageal echocardiography is integral to identifying and risk stratifying this complication, and imagers should have a low threshold to screen for it, particularly in the setting of haemodynamic compromise. The decision to deploy a closure device should be made on a case-by-case basis considering the risks and benefits of doing so compared with conservative management.

**Learning points**

- To identify risk factors associated with interatrial dissection.
- To recognize complications of transseptal punctures quickly.
- To demonstrate the use of closure devices in transseptal puncture complications.

## Introduction

Mitral valve (MV) transcatheter edge-to-edge repair (TEER) is an effective therapeutic option for patients with prohibitive operative risk who remain symptomatic from primary or secondary mitral regurgitation (MR) despite optimal medical therapy.^1^ Access to the MV during a TEER procedure is commonly facilitated through interatrial transseptal puncture (TSP). This controlled trauma inherently increases the risk of unplanned damage to the interatrial septum (IAS), although complications are rare in the contemporary era with a reported incidence of <1%.^2,3^

We present a case of an 85-year-old woman with severe MR resulting in heart failure hospitalizations who underwent a MV TEER procedure. This was complicated by an iatrogenic IAS dissection and subsequently managed with an Amplatzer Septal Occluder (Abbott Cardiovascular). Although this complication has historically been managed conservatively,^2–6^ we believe this case outlines the role of closure devices in select circumstances.

## Summary figure

**Figure:**
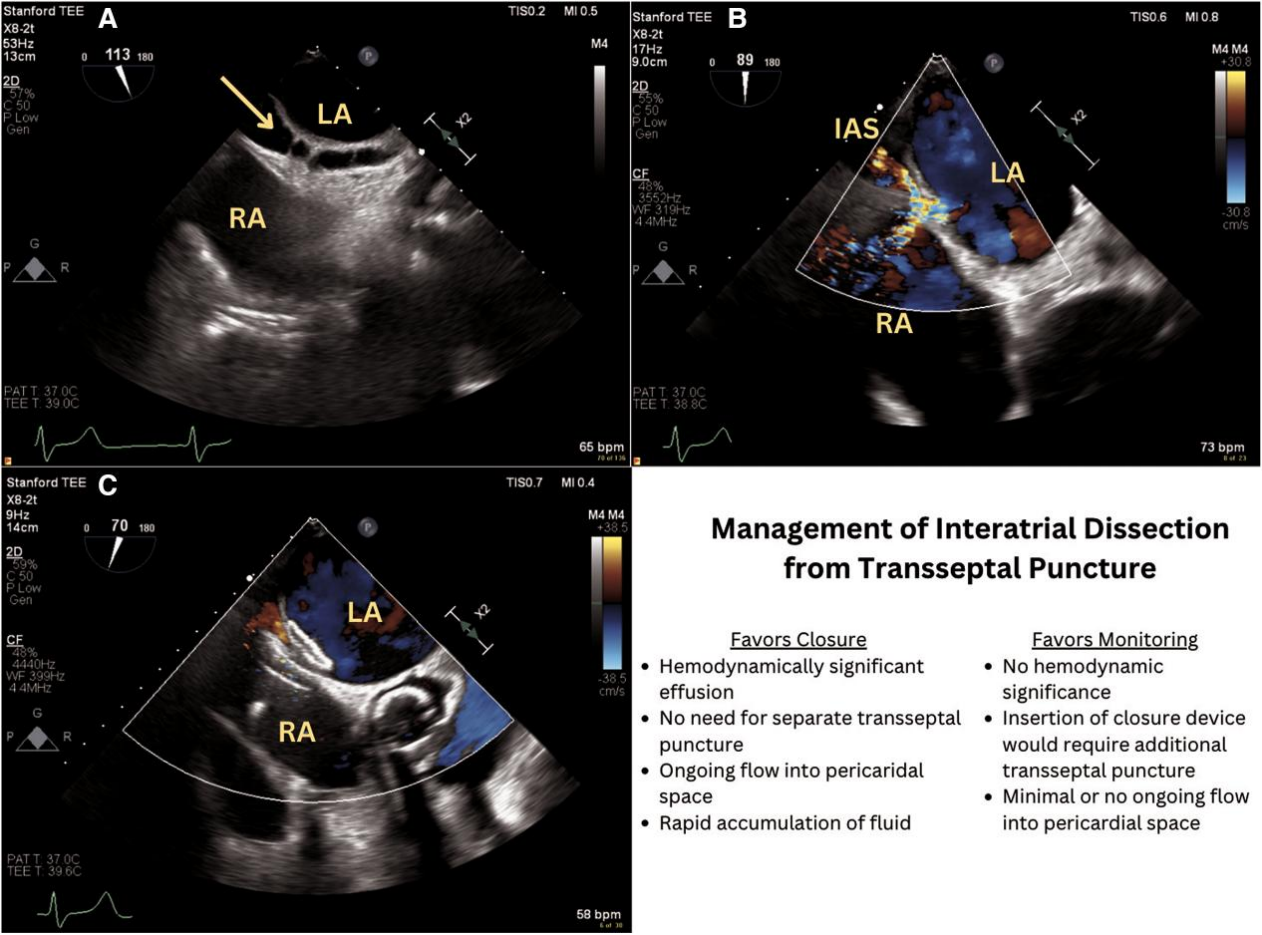
(*A*) Identification of interatrial dissection on transoesophageal bi-caval view (yellow arrow). (*B*) Flow from the left atrium into both the right atrium and interatrial dissection on modified bi-caval view. (*C*) Reduction of flow from LA into RA and IAS following placement of Amplatzer closure device across defect on modified bi-caval view. IAS, interatrial septum; LA, left atrium; RA, right atrium.

## Case presentation

Our patient’s cardiac history included heart failure with preserved ejection fraction and severe aortic stenosis managed 3 years prior with transcatheter aortic valve replacement (TAVR). A second overlapping 23 mm Sapien3 (Edwards Lifesciences) valve was implanted during the index TAVR procedure to manage her aortic stenosis and provide stabilization when the initial TAVR embolized towards the aortic root. Her MR was graded as moderate at the time of TAVR. After an initial improvement in symptoms, she had recurrence of exertional dyspnoea and a heart failure hospitalization 6 months prior to her MV TEER procedure associated with the progression of her MR to severe. Her extracardiac history included chronic steroidal immunosuppression for over 20 years following a diagnosis of microscopic polyangiitis leading to renal transplantation. Her pre-operative physical exam was notable for friable skin, with a subcutaneous haematoma caused by standard blood pressure cuff deployment. Her cardiac exam revealed normal heart sounds, a Grade 2 mid-peaking systolic crescendo–decrescendo murmur heard best at the right upper sternal border and a Grade 2 holosystolic murmur heard best at the apex.

**Figure 1:**
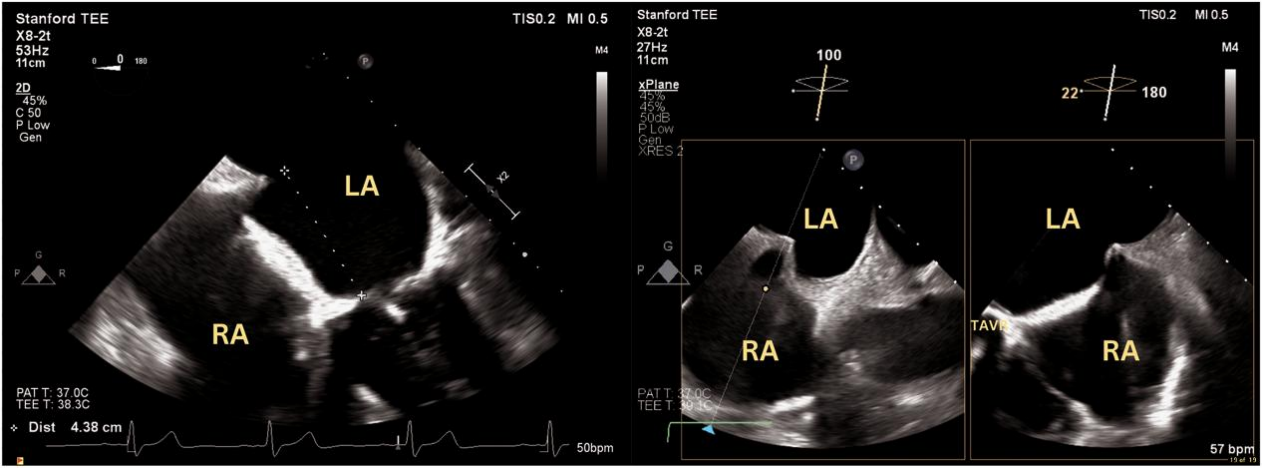
Transseptal puncture height of 4.4 cm above mitral valve annulus at the mid-posterior aspect of interatrial septum, standard positioning for a mitral valve transcatheter edge-to-edge repair procedure. LA, left atrium; RA, right atrium; TAVR, transcatheter aortic valve replacement.

Pre-operative transoesophageal echocardiogram (TEE) confirmed severe MR and a structurally normal IAS (see Supplementary material online, *Video S1*). Under real-time fluoroscopic and TEE guidance, a TSP was performed using a VersaCross radiofrequency wire (Baylis Medical). A single puncture was performed in the standard position for MV TEER procedure (*Figure 1*). The PASCAL Precision steerable guide sheath and PASCAL ACE implant (Edwards Lifesciences) were subsequently delivered across the septum. No IAS morphological changes were appreciated immediately post-puncture or while observing the guide sheath through the IAS.

**Figure 2:**
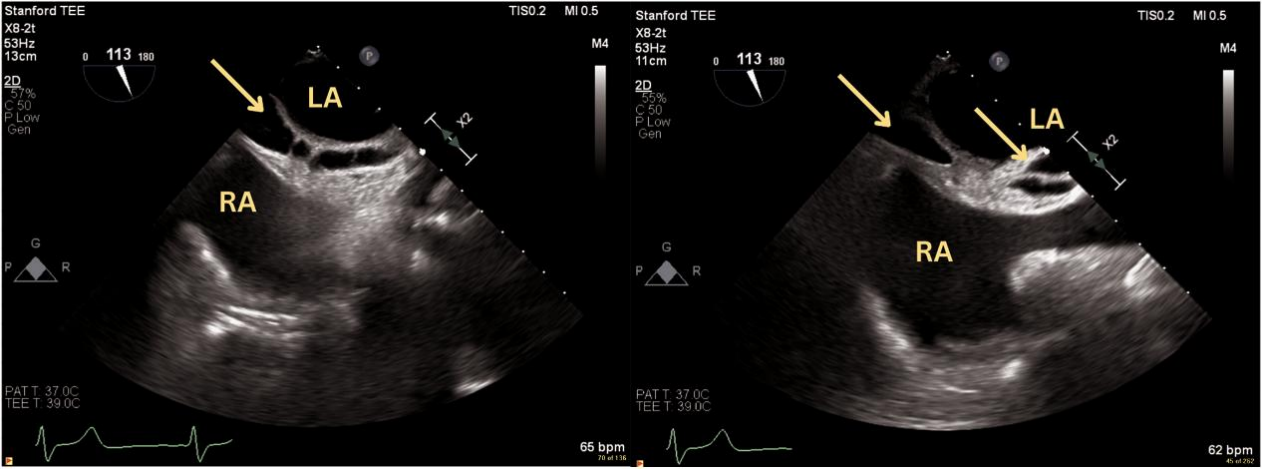
Following initial mitral valve transcatheter edge-to-edge repair device placement, a small, organizing effusion was noted within the interatrial septum, extending inferior to superior (arrows) as seen in the transoesophageal echocardiogram bi-caval view. LA, left atrim; RA, right atrium.

**Figure 3:**
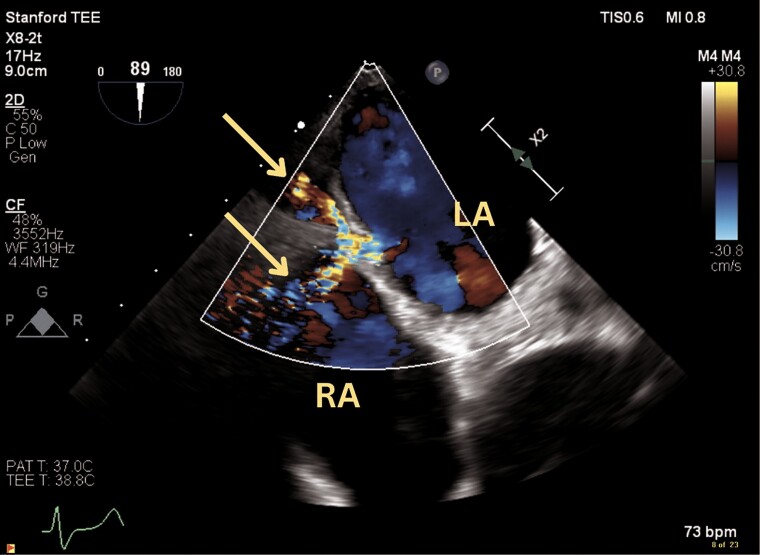
Modified bi-caval transoesophageal echocardiogram view with colour Doppler demonstrating flow from the left atrium into the interatrial septum dissection, pericardial space and right atrium (arrow). LA, left atrium; RA, right atrium.

**Figure 4:**
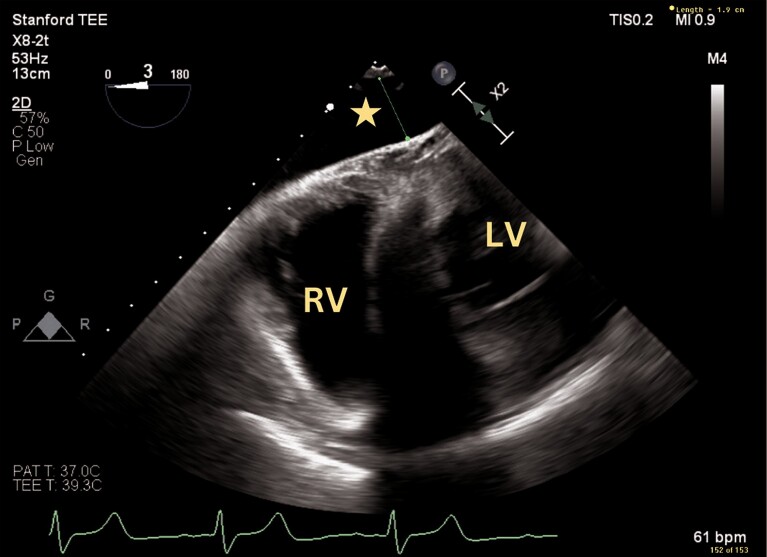
Identification of pericardial effusion (star) in gastric transoesophageal echocardiogram view. LV, left ventricle; RV, right ventricle.

During the interrogation of the residual moderate MR following the initial grasp attempt, a small, organizing effusion was noted within the IAS (*Figure 2*; Supplementary material online, *Video S2*). Further evaluation revealed an IAS dissection originating inferior to the TSP and extending superiorly (*Figure 2*; Supplementary material online, *Video S2*). The inferior aspect of the dissection coursed along the inferior left atrial wall with flow into the pericardial space (*Figure 3*; Supplementary material online, *Video S3*) resulting in a pericardial effusion (*Figure 4*; Supplementary material online, *Video S4*).

Differential diagnoses for peri-procedural pericardial effusion include right or left atrial free-wall puncture, ventricular puncture, or caval injury. Most of these diagnoses can be quickly ruled in or out by echocardiographic imaging.

**Figure 5:**
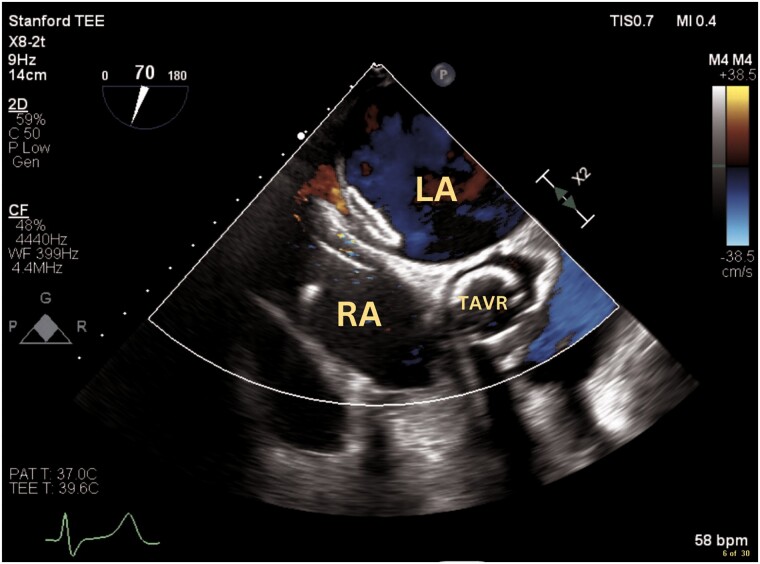
A 30 mm Amplatzer Occluder device was deployed across the interatrial septum, with subsequent reduction of flow across the interatrial dissection. LA, left atrium; RA, right atrium; TAVR, transcatheter aortic valve replacement.

In the setting of cardiac tamponade and improvement in MR severity, the MV TEER device was deployed. A subxiphoid pericardiocentesis was performed with an 8-Fr pigtail catheter with an immediate improvement in haemodynamics. The 0.035 in PASCAL Precision guide sheath was exchanged for a 12 Fr Amplatzer delivery sheath (Abbott Cardiovascular), and a 30 mm Amplatzer Septal Occluder was then deployed across the TSP site. This resulted in a visible reduction of flow into the IAS dissection (*Figure 5*; Supplementary material online, *Video S5*).

Transthoracic echocardiogram on post-operative Day 2 showed no re-accumulation of effusion. Transthoracic echocardiogram at 1-month post-deployment showed reduced MR, a well-seated septal occluder device, and no significant re-accumulation of pericardial fluid.

## Discussion

Most transcatheter-related interatrial dissections have been observed peri-procedurally or early in the post-procedural course.^2,5,7^ Underlying risk factors for iatrogenic IAS damage include older age, multiple TSP attempts, and increased IAS thickness.^2,8,9^ Our patient’s fossa ovalis thickness was mildly increased; however, the singular TSP attempt was still performed on the thinnest portion of the septum. Chronic steroid use is not known to be an established risk factor, but in the absence of other notable issues, we wonder if this may have been a contributing factor.

Transoesophageal echocardiogram is critical in identifying and guiding the management of TSP complications. In retrospect, early TEE signs of IAS dissection may have been present soon after crossing the IAS, prior to bringing the TEER device below the level of the valve (see Supplementary material online, *Figure S1*). These findings are subtle and are outside the immediate area of interest. Optimizing image resolution for procedures often involves limiting extraneous structures. Conversely, monitoring for complications inherently requires visualization of extravalvular structures. Imaging providers must, therefore, have a high degree of sensitivity for identifying complications that may arise throughout a procedure. Having a low threshold to assess for complications with TEE, particularly if the patient is demonstrating haemodynamic changes, is essential to early identification of complications.

There is currently no consensus on how to best manage iatrogenic interatrial dissections. Of the previous case reports available, all favoured observation over intervention.^2–6^ In these cases, patients remained haemodynamically stable, and for some, complications were only identified after the procedure concluded. Our decision to close the communication was made due to the haemodynamic consequences of a growing pericardial effusion and ongoing blow flow into the pericardial space. We felt the risk of ongoing extravasation outweighed the risk of further IAS trauma, particularly as the PASCAL guide remained in place across the septum, eliminating the need for a separate TSP for septal occluder placement.

To our knowledge, this is the first reported instance of using a septal occluder device in treating an IAS dissection related to TSP. Transoesophageal echocardiogram is integral to identifying this uncommon, but significant complication and chronic steroid use may be an underappreciated risk factor. The decision to close an IAS dissection should be made on a case-by-case basis considering a patient’s clinical status and the risks and benefits of intervention or observation.

## Supplementary Material

ytae559_Supplementary_Data

## Data Availability

No new data were generated or analysed in support of this research.

## Lead author biography

Dr Isabel Kim completed her medical school at McMaster University in Hamilton, Ontario before pursuing both her internal medicine residency and cardiology fellowship at the University of British Columbia in Vancouver. She is currently completing a 1-year fellowship in advanced structural imaging with a special focus on echocardiographic guidance of structural heart procedures at Stanford University. Her clinical and academic interests include valvular heart disease, transcatheter structural interventions, and medical education.

## Supplementary material

**Consent:** The patient provided written consent for discussion and dissemination of images used in her clinical case, adherent with current COPE guidelines.

**Funding:** The author(s) received no financial support for the research, authorship, and/or publication of this article.
