# Supplementary material for: Interatrial septum dissection and closure from transseptal puncture during mitral transcatheter edge-to-edge repair: a case report
Source: Eur Heart J Case Rep. 2024 Nov 2;8(11):ytae559. doi: 10.1093/ehjcr/ytae559 (PMC11561578; doi:10.1093/ehjcr/ytae559)
Supplement: ytae559_Supplementary_Data [file ytae559_supplementary_data.zip › Supplemental Figure Video Legends.docx]

Supplemental Figures and Video Legends

**Supplemental Video 1:** Baseline, pre-procedural intact interatrial septum as seen in the transesophageal bicaval TEE view. *TEE=transesophageal echocardiogram.*

**Supplemental Video 2:** Bi-caval sweep on TEE showing the interatrial dissection across superior to inferior aspect of interatrial septum. *TEE=transesophageal echocardiogram.*

**Supplemental Video 3:** Modified bi-caval view with color Doppler on TEE demonstrating flow from the LA into the IAS dissection, the pericardial space and RA (yellow arrows). *IAS=interatrial septum; LA=left atrium; RA=right atrium; RV= right ventricle; TEE=transesophageal echocardiogram.*

**Supplemental Video 4:** Gastric view on TEE showing hemodynamically significant pericardial effusion posterior to the cardiac chambers. *TEE=transesophageal echocardiogram.*

**Supplemental Video 5:** Modified bi-caval view showing the 30mm Amplatzer Occluder device deployed across the interatrial septum, with subsequent reduction of flow across the interatrial dissection.

**Supplement Figure 1:** Bi-commissural view with lateral X-plane to long-axis TEE view possibly showing early signs of interatrial dissection prior to bringing the PASCAL ACE implant below the level of the valve (yellow arrow). *LA= left atrium; LV=left ventricle; TAVR=transcatheter aortic valve replacement; TEE=transesophageal echocardiogram*
