# Supplementary figures and images for: Interatrial septum dissection and closure from transseptal puncture during mitral transcatheter edge-to-edge repair: a case report
Source: Eur Heart J Case Rep. 2024 Nov 2;8(11):ytae559. doi: 10.1093/ehjcr/ytae559 (PMC11561578; doi:10.1093/ehjcr/ytae559)

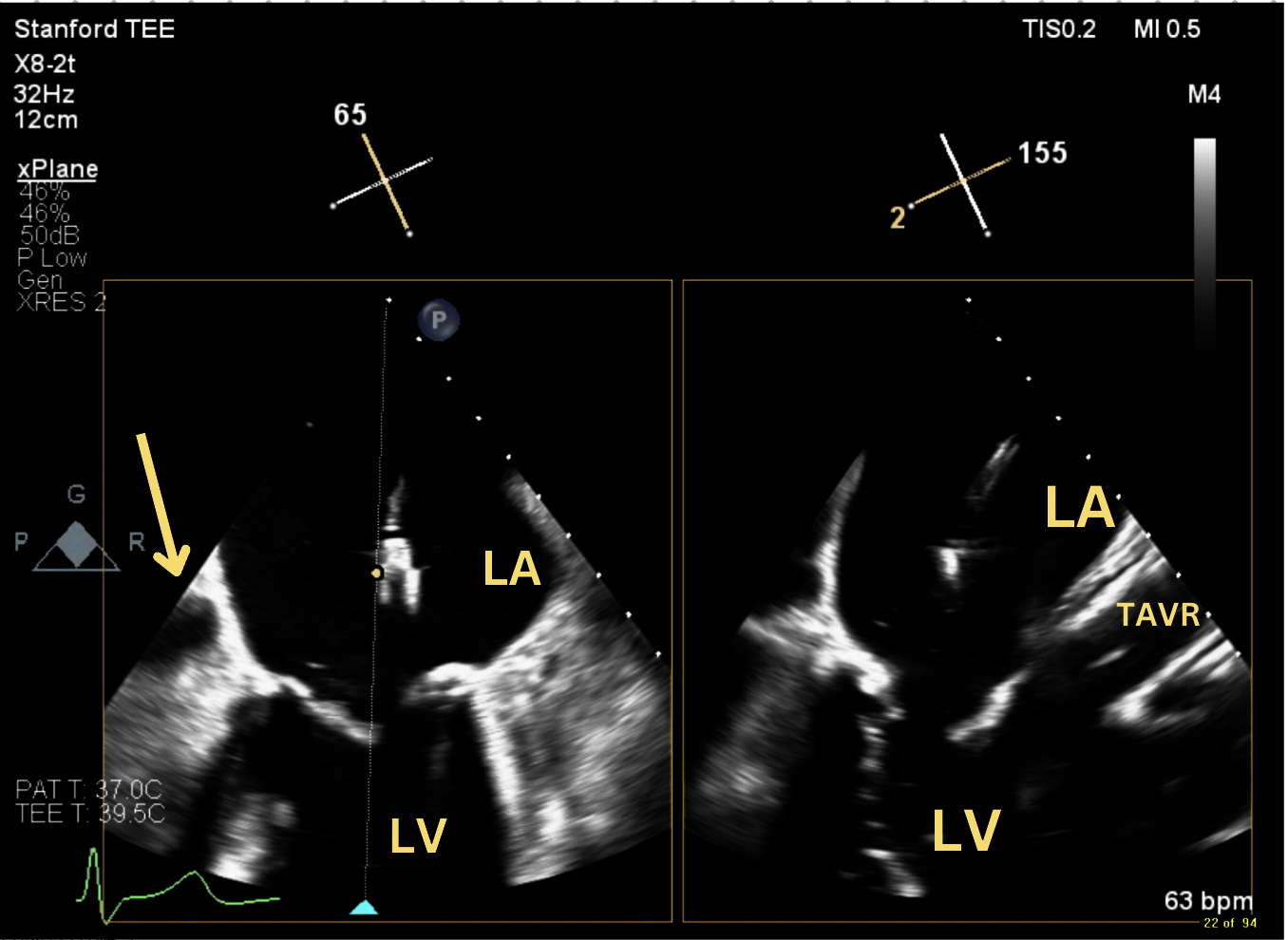

Supplement: ytae559_Supplementary_Data [file ytae559_supplementary_data.zip › Supplement_Figure_1_400_cmyk.jpg]
